# Genome-scale metabolic modeling uncovers cell-type specific signatures associated with APOE variants

**DOI:** 10.1016/j.isci.2026.115638

**Published:** 2026-05-07

**Authors:** Dilara Uzuner Odongo, Roxan A. Stephenson, Linling Cheng, Linda G. Yang, Priyanka S. Narayan, Tunahan Çakır, Madhav Thambisetty

**Affiliations:** 1 Department of Bioengineering, Gebze Technical University, Gebze, Kocaeli, Turkey; 2 Genetics and Biochemistry Branch, National Institute of Diabetes and Digestive and Kidney Diseases, National Institutes of Health, Bethesda, MD, USA; 3 Intramural Research Program, National Institute on Aging, National Institutes of Health, Baltimore, MD, USA

**Keywords:** Systems biology, Omics

## Abstract

Metabolic dysregulation is a key feature of Alzheimer’s disease (AD) pathogenesis, with the APOE ε4 variant (APOE4) representing the strongest genetic risk factor. In this study, we utilized a metabolite-centric approach to investigate how APOE4 reshapes cellular metabolism across brain cell types. Transcriptomic data from isogenic iPSC-derived neurons, astrocytes, and microglia were integrated into a human genome-scale metabolic model to identify genotype-specific alterations. These findings were validated using metabolomics data from the same cell types. In addition to cholesterol and fatty acid dysregulation, we identified alterations in bile acid biosynthesis, folate metabolism, and thyroid hormone metabolism. Similar metabolic signatures were also detected in human postmortem transcriptomic data. Integrating transcriptomic and metabolomic data enhances the understanding of biological mechanisms underlying APOE4-associated metabolic dysregulation in AD.

## Highlights

• APOE4 drives cell-type specific metabolic reprogramming in neurons and glia
• Bile acid, folate, and thyroid metabolism are APOE4-dysregulated pathways
• Multi-omics integration identifies distinct metabolic signatures of APOE-genotype
• Postmortem human data validates the metabolic profiles from iPSC-derived cells

## Graphical abstract

**Figure:**
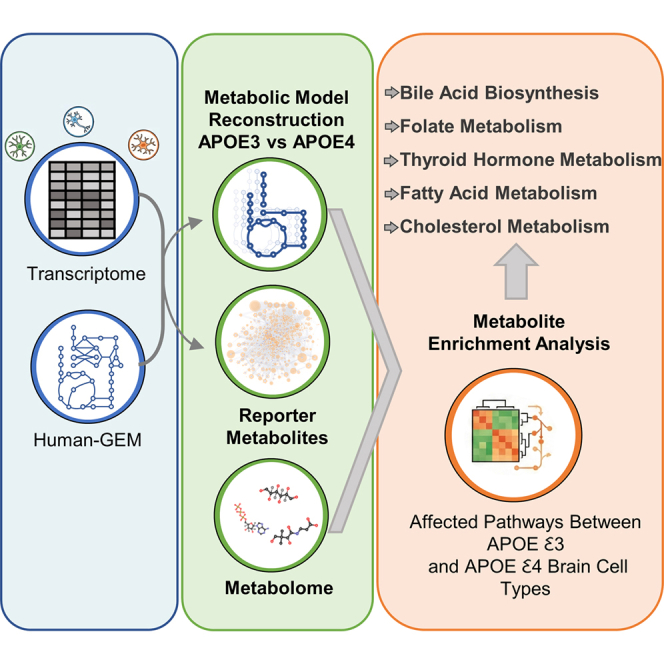

## Introduction

Recent advances in high-throughput technologies have led to a substantial increase in availability of multi-omics datasets. This presents a new challenge; how can researchers integrate these datasets to comprehensively understand cell biology? Genome scale metabolic models (GEMs) provide the list of all known metabolic reactions and their associated genes in an organism and enable simulations of metabolic phenotypes.^1^^,^^2^ They are commonly used to investigate diseases^3^^,^^4^ and identify biomarkers^5^^,^^6^ or drug targets.^7^^,^^8^ It is also possible to integrate transcriptome data with GEMs^9^^,^^10^^,^^11^^,^^12^^,^^13^ to better characterize the underlying metabolic phenotypes of diseases. Therefore, they can serve as scaffolds for metabolism-centered integration of multi-omics data.

The ε4 allele of the *APOE* gene is the strongest known genetic risk factor for late-onset AD. The single amino acid difference between the *APOE*3 and *APOE*4 coding sequence (C112R)^14^^,^^15^ alters composition of cellular lipids as well as susceptibility to amyloid beta plaque accumulation.^14^^,^^16^ However, our understanding of the effects of *APOE* gene variants on cellular metabolism remains incomplete. While the predominant focus on biological mechanisms underlying AD has centered around the amyloid hypothesis, we now recognize that changes in metabolism play an important role in the disease pathology.^17^^,^^18^^,^^19^

In this study, we applied a metabolite-centric approach to analyzing transcriptome and metabolome datasets from APOE3 and APOE4 neurons, astrocytes and microglia derived from an isogenic set of human iPSCs.^20^ First, we used transcriptomic data to construct cell-type specific metabolic networks for each genotype based on a human GEM,^21^ which revealed altered metabolic reactions between *APOE3* and *APOE4*-expressing cells in each cell type. Metabolites of the altered reactions were then used to identify altered metabolic pathways for each cell type. As a complementary approach, we mapped transcriptomic data onto the human GEM using a graph-based algorithm^22^ to determine reporter metabolites and associated altered pathways. Finally, the data of the same cell types were additionally analyzed to identify pathways consistently altered at both transcriptome and metabolome levels. Other studies of iPSC-derived human cells have focused on changes in lipid metabolites exclusively.^23^ This broad approach to understand *APOE* variant-associated global disruptions to cellular metabolism demonstrates how a multi-omics dataset in the form of transcriptome and metabolome data can be systematically integrated to identify *APOE* genotype-associated metabolic pathway perturbations.

## Results

### Reconstruction of cell-type specific metabolic networks in neurons, astrocytes, and microglia reveals APOE3 versus APOE4 differences

**Figure 1:**
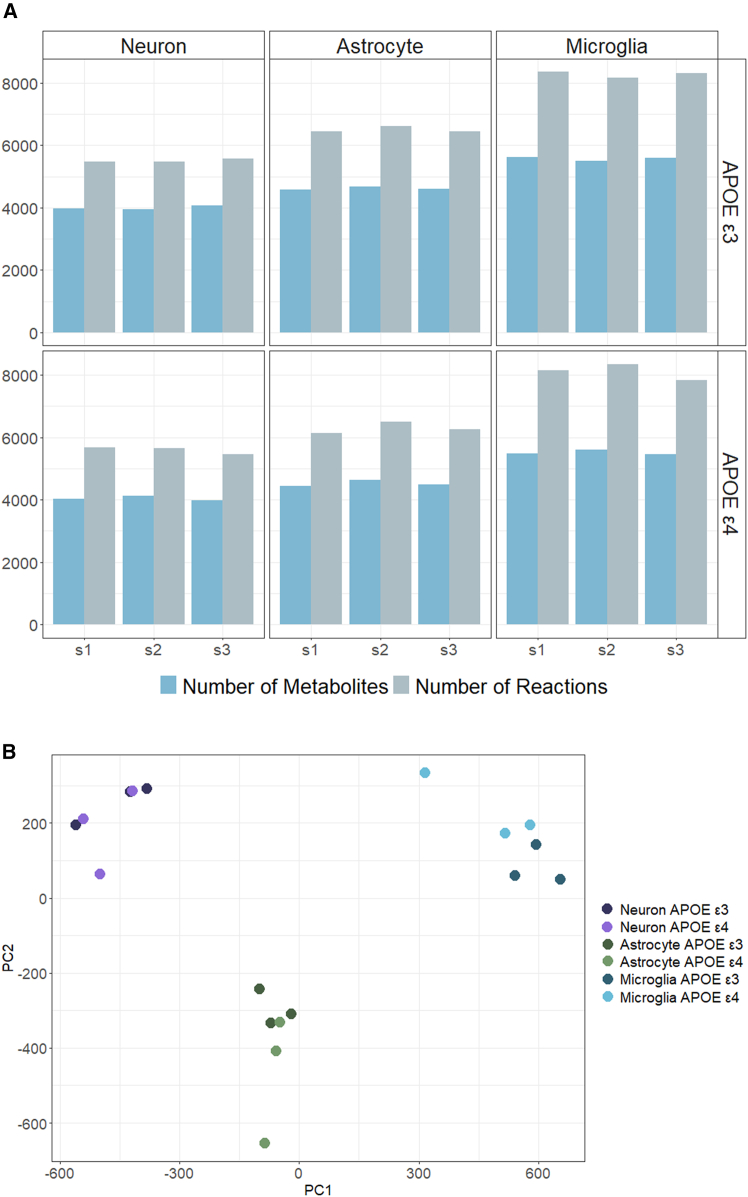
Characterization of the iMAT models across the cell-types and APOE status (A) Number of active reactions and metabolites in the iMAT-derived metabolic models for each sample (s1-s3 in *x* axis shows different samples). (B) PCA graph of the binarized iMAT models. The proportion of deviance explained by this model is 66%.

Transcriptomic data of iPSC derived *APOE3* or *APOE4* carrying neurons, astrocytes,^20^ and microglia were used to construct metabolic models of each sample by the iMAT algorithm. iMAT^9^ is commonly used to identify metabolic alterations from human disease-associated transcriptome datasets.^24^^,^^25^^,^^26^^,^^27^ Here, we integrated the transcriptome data of each sample with the human genome scale metabolic model Human-GEM, and used iMAT to predict active/inactive metabolic reactions. While the number of active reactions and metabolites shows clear cell type-specific patterns, as shown in Figure 1A, there is no considerable difference in the number of active reactions or metabolites based on cell-types between *APOE3* and *APOE4* samples. Principal component analysis (PCA) of the binarized vectors representing sample-specific metabolic networks (active reactions as 1, inactive as 0) reveals discrimination between the cell types based on reaction content, indicating that the iMAT-reconstructed metabolic models effectively capture cell type-specific metabolic profiles (Figure 1B). Overall, these results suggest that cell type-specific models utilize different active metabolic routes while at a global level, *APOE3* and *APOE4* genotypes are relatively similar.

**Figure 2:**
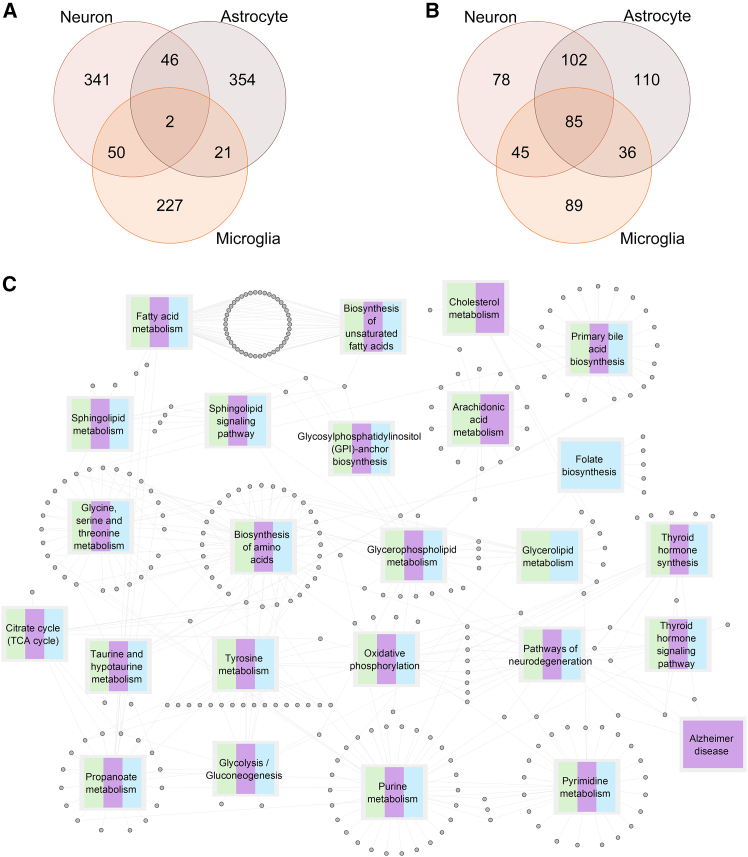
Reaction, pathway and metabolite level results of the iMAT analysis Number of altered reactions and pathways for each cell type between APOE ε3 and ε4 states. (A) Venn diagram showing the number of altered reactions in each cell type based on the iMAT models. See Table S1 for the full list. (B) Venn diagram showing the number of metabolites consumed/produced by the altered reactions in each cell type based on the iMAT models. (C) Selected 25 metabolite-based pathway enrichment terms. See Table S2 for the full list. Gray nodes represent matching metabolites. Rectangular nodes represent enriched pathways. Each pathway is colored according to the cell type it is enriched in. Neuron: green, astrocyte: pink and microglia: blue.

To further assess differences between *APOE3* and *APOE4* models, the altered reactions between the two conditions were investigated. A total of 439 reactions from neuron models, 423 reactions from astrocyte models and 300 reactions from microglia models were predicted to be altered in *APOE3* or *APOE4* genotypes. The robustness of the identified metabolic alterations was further assessed through statistical validation. These analyses indicated that the identified perturbed reactions are associated with the APOE genotype rather than stochastic network noise (Figure S2). Next, reaction activity/inactivity information was compared between the genotypes (Figure 2A; Table S1). Most of the identified reactions were found to be cell-type specific (Figure 2A). To document functional effects of the altered reactions, we followed a metabolite-centric approach by focusing on the metabolites of the altered reactions (Figure 2B). Compared to the altered reactions (overlap coefficient *p* value = 0.04), their associated metabolites have considerably higher overlap (overlap coefficient *p* value = 1.57 × 10^−182^) between the cell types. In other words, although the reaction contents in cell-specific models are not significantly similar between cell types, the metabolites used or produced in these reactions show significant intercellular similarity. We then identified KEGG pathways significantly enriched with these metabolites, using MBROLE, yielding 145, 192, and 179 pathways (*p* value <0.05) for neuron, astrocyte and microglia, respectively, between *APOE3* and *APOE4* genotypes (Table S2). Figure 2C and Data S1 shows 25 selected enriched pathways and the cell types in which they are enriched.

Pathways enriched with the metabolites of the reactions altered between *APOE3* and *APOE4* were rigorously analyzed to demonstrate the feasibility of our approach. A significant number of pathways were altered across all three cell types, including the biosynthesis of amino acids (specifically glycine, serine, threonine, and tyrosine metabolism), core energy production pathways (glycolysis, TCA cycle, and oxidative phosphorylation), and several lipid-related pathways such as bile acid biosynthesis, glycerophospholipid metabolism, GPI-anchor biosynthesis, and sphingolipid metabolism. Additionally, thyroid metabolism was altered across all three cell types. In a more specific pattern, pathways for fatty acid metabolism, fatty acid biosynthesis, and cholesterol metabolism were uniformly altered in two cell types: neurons and astrocytes. Finally, some metabolic perturbations were unique to a single cell type. Arachidonic acid metabolism was altered only in astrocytes, while folate metabolism was specifically altered in microglia.

These findings are in strong agreement with existing literature on the distinct biochemical functions of the *APOE* isoforms. For example, the apoE protein is a primary cholesterol transporter in the brain, with the *APOE4* isoform associated with reduced efficiency in cholesterol transport in neurons and higher cholesterol synthesis and accumulation in astrocytes.^28^^,^^29^ Our results also align with findings that sterol and sphingolipid metabolite levels are altered in the brains of *APOE4*+ AD patients.^30^ This fundamental APOE-driven mechanism is highly relevant to AD, as widespread lipid dysregulation is a hallmark of the disease. Especially, in neurons and astrocytes, fatty acid metabolism and fatty acid biosynthesis pathways are dramatically enriched. This is reminiscent of previous studies in AD patients and human iPSC-derived cells, which suggest a role for fatty acid biology.^31^^,^^32^^,^^33^^,^^34^ Glycerophospholipids are complex fatty acid species, and they are the main lipid class in the brain.^32^^,^^35^^,^^36^^,^^37^ Arachidonic acid is a member of omega-6 fatty acid family, and it has pro-inflammatory effect and increases in AD brains.^23^^,^^32^^,^^38^^,^^39^^,^^40^^,^^41^ Bile acid biosynthesis was reported to be altered in the brains of AD patients^42^^,^^43^ although a direct association with the *APOE* genotype is missing in the literature.

There are many publications in the literature about the relationship of amino acid metabolism with AD.^44^^,^^45^^,^^46^^,^^47^ L-serine biosynthesis has been previously reported to be reduced in AD brain suggesting that its supplementation might be a potential therapy for AD.^48^ Glycine, serine and threonine metabolism was reported to be important for brain energy production, which is interrupted in AD.^49^ Tyrosine metabolism is also important in the synthesis of several neurotransmitters including epinephrine, norepinephrine, and dopamine. As a supportive result, we observed significant enrichment in the KEGG term “Pathways of Neurodegeneration” in all three cell types which covers molecular mechanisms across multiple neurodegenerative diseases. Additionally, “Alzheimer disease” KEGG pathway is enriched only in astrocytes.

Changes in the efficiency of energy production related metabolic pathways including glycolysis, TCA cycle, and oxidative phosphorylation may contribute to signaling and transcriptional defects associated with neurodegenerative diseases.^50^^,^^51^^,^^52^ Also, prior studies showed that glycolysis efficiency was increased in astrocytes and decreased in microglia of *APOE4* genotype cells.^53^^,^^54^ These results may help elucidate the cellular mechanisms by which the APOE4 genotype contributes to an elevated risk for AD.

Finally, our study highlights pathways where the direct link to the *APOE* genotype itself has been less defined. For instance, while both low folate levels and thyroid dysfunction are recognized as treatable causes of cognitive impairment and risk factors for cognitive decline and AD,^55^^,^^56^^,^^57^^,^^58^ a direct, mechanistic link to specific *APOE* isoforms has not been well characterized. Our findings that folate metabolism (in microglia) and thyroid metabolism (in all cell types) are altered in an *APOE*-dependent manner may therefore represent a previously uncharacterized connection, suggesting a potential mechanism through which the *APOE4* genotype could influence these crucial aspects of brain health.

### Graph-based transcriptome mapping complements mass-balance based results by uncovering AD-associated pathways

Reporter metabolite analysis (RMA) is a graph-theoretical data analysis technique to reveal the transcriptional regulatory architecture of metabolic networks. It uses topological information of the metabolic models and transcriptome data to identify metabolites around which the most significant transcriptional changes occur.^22^ The algorithm transforms gene expression information to metabolite information, and it was used to predict sources of metabolic perturbations in several diseases.^3^^,^^10^^,^^11^^,^^12^

**Figure 3:**
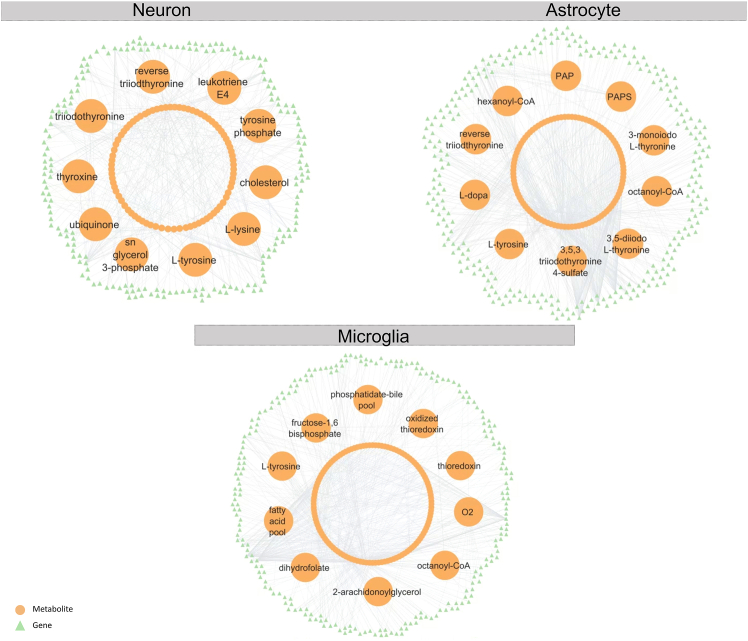
Reporter metabolites Orange balls represent the reporter metabolites and green triangles represent the neighbor genes. Totally 90, 108, and 130 metabolites for neurons, astrocytes and microglia-like cells were found as reporter metabolites. The top ten metabolites that have the lowest *p* values were highlighted in the figure for each cell type.

RMA was applied to the transcriptome data by using the topology of the metabolic networks as scaffolds to identify dysregulated metabolites in *APOE4* condition in each cell type. RMA approach scores metabolites based on the *p* values of the surrounding genes. 90, 108, and 130 metabolites for neurons, astrocytes and microglia-like cells were found as reporter metabolites, respectively (Figure 3; Table S3). The amino acids L-tyrosine and tyrosine-phosphate were found to be reporter metabolites common to all cell types. Tyrosine metabolism was also found in the metabolite-based pathway enrichment of the iMAT results. Similarly, thyroxine and triiodothyronine in neurons and 3,5-diiodo-L-thyronine and 3,5,3-triiodothyronine-4-sulfate in astrocytes, (identified also in the iMAT metabolite-based pathway enrichment), were identified as reporter metabolites. The dopamine precursor L-DOPA was identified as a reporter metabolite in astrocytes while dopamine itself was identified in neurons (Table S3). Oxygen and thioredoxin are reporter metabolites in microglia. Multiple studies have reported an association between thyroid disorders and neurodegenerative diseases including AD.^59^^,^^60^^,^^61^^,^^62^^,^^63^ It is known that abnormal thyroid function is a risk factor for Alzheimer’s disease.^64^ Also, thyroid hormone related actions are performed by 3,5,3-triiodothyronine in neurons, which is produced from thyroxine in astrocytes.^63^^,^^65^ It has been shown that in plasma of AD patients L-DOPA/tyrosine ratio is significantly higher than healthy individuals while the dopamine/L-DOPA ratio is significantly lower.^66^ Oxygen and thioredoxin play roles in redox reactions and mitochondrial metabolism. Thioredoxin is involved in signaling pathways associated with AD.^67^ In neurons, the reporter metabolite ubiquinone is known to reduce reactive oxygen species.^68^

As mentioned previously, several reporter metabolites identified by RMA are related to AD. However, it is important to understand these changes at the pathway level to understand the overall pattern. To this end, similar to the iMAT metabolites, enrichment analysis was applied to the reporter metabolites (Table S2). Thyroid hormone, bile acid, and oxidative phosphorylation pathways were enriched in both neurons and microglia. Cholesterol metabolism is enriched in neurons. The KEGG term “pathways of neurodegeneration” is enriched in all three cell types. Fatty acid metabolism terms are enriched in both astrocytes and microglia. When comparing the analyses, the terms identified using iMAT metabolites were generally similar to the terms identified using reporter metabolites. However, their distribution across cell types differed: with iMAT, more terms were shared among the three cell types, a pattern that was not observed with RMA.

Both iMAT and reporter metabolite analyses integrate gene expression data with a GEM, yet they differ methodologically. iMAT is a mass-balance-based integration method that aims to identify metabolic reactions active in a given sample by optimizing consistency between expression data and predicted reaction activity (flux). In contrast, RMA is a graph-based integration method to highlight metabolites that are surrounded by reactions showing coordinated-changes in the expression of their genes, without predicting reaction fluxes. Mass-balance based framework of iMAT accounts for possible post-transcriptional regulations indirectly by using gene expression data to infer reaction activity (flux) while RMA incorporates gene-level *p* values directly into its calculation. Our results indicate that these two analyses produce overlapping results despite their methodological differences while they also have complementary results in a cell-type specific context. Hence, the combined use of mass-balance based and graph-based integration methods enhances the comprehensiveness of the results.

### Metabolome data confirms dysregulated pathways identified by transcriptome data analysis

As a complementary approach, metabolome data generated from the same *APOE3* or *APOE4* neurons, astrocyte, and microglia were analyzed. Metabolites with significantly altered levels between *APOE3* and *APOE4* samples were identified (Table S4). Accordingly, the numbers of metabolites with significant changes in their levels were 44, 38, and 5 for neurons, astrocytes, and microglia, respectively.

**Figure 4:**
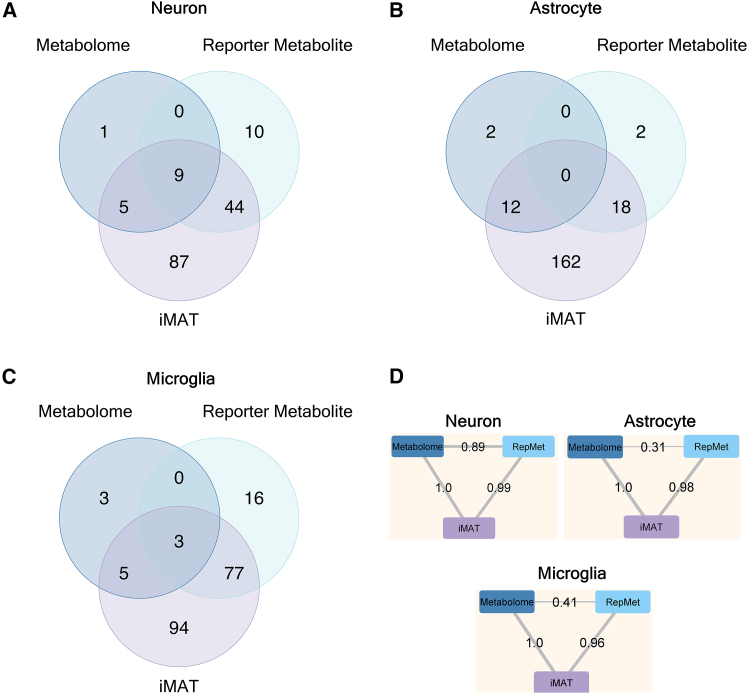
Metabolite-based pathway enrichment results (A–C) Number of enriched pathways (*p* value <0.05) for (A) neuron, (B) astrocyte, and (C) microglia cells by iMAT and RMA, the transcriptome-based methods that incorporate a genome-scale metabolic model. Metabolites from iMAT-identified altered reactions, and reporter metabolites from RMA were each used for pathway enrichment as well as the differentially abundant metabolites from metabolome data. (D) Overlap coefficients between the pathway annotations of the metabolites identified by the three approaches.

We asked whether the altered pathways captured by transcriptome mapping on the human genome-scale metabolic network were also reflected in metabolome-based differential analysis. To this end, first, metabolites with significantly altered levels based on the metabolome data were subjected to pathway enrichment analysis using the MBROLE database. Similarly, enrichment analysis was also applied to the metabolites obtained in the iMAT-based altered reactions (also presented in the first section of results) and the metabolites identified with the graph-based RMA (also presented in the second section of results). Figures 4A–4C shows the number of common and unique enriched pathways for each method (full list of the enrichment results is in Table S1). In neurons, 9 pathways were found to be commonly enriched with the metabolites from the three approaches; differential metabolome data, iMAT reactions, and RMA. In astrocytes, 12 and 18 pathways were found to be common between iMAT and metabolome analyses and iMAT and reporter metabolite analyses, respectively, while no terms were found to be common between metabolome and reporter metabolite analyses. In microglia, 3 pathways were found to be commonly enriched with the metabolites from the three approaches. 8 out of 11 pathways of the metabolome enrichment were common with the iMAT reactions.

In neurons, the common pathways enriched in all three analyses, and, hence, predicted to be altered between *APOE3* and *APOE4* genotypes, include primary bile acid biosynthesis,^42^ biosynthesis of amino acids,^45^^,^^47^ and glycine, serine, and threonine metabolism,^48^ which were previously reported to be associated with AD. In astrocytes, common pathways enriched with the iMAT derived metabolites, and the differential metabolite levels include sphingolipid signaling pathway, glycerophospholipid metabolism, primary bile acid metabolism, and cholesterol metabolism. Metabolite enrichment results emerging from both iMAT and RMA approaches include fatty acid metabolism, purine metabolism and pathways of neurodegeneration in astrocytes. For microglia, bile acid biosynthesis and retrograde endocannabinoid signaling are common in all three analyses. Additionally, KEGG term “*Escherichia coli* infection” is also common in all three analyses. These findings align with recent research indicating that lipopolysaccharide treatment of microglia induces lipid metabolic changes similar to those observed in microglia carrying the APOE4 genotype. GPI-anchor biosynthesis is common between iMAT and metabolome results. KEGG terms fatty acid metabolism, thyroid hormone synthesis, and pathways of neurodegeneration are common between iMAT and reporter metabolites.

In addition, the metabolites captured from three different sources (differential metabolome, iMAT-based altered reactions and RMA) were compared in terms of the similarity of their pathway associations. This also enabled comparison of results for the cell-types with a low number of significantly altered metabolites, e.g., microglia. The results revealed that, although the number of common terms obtained as a result of enrichment were low as discussed previously, the pathway annotations of the metabolites between the differential metabolome analysis and the transcriptome-derived metabolic network approaches were similar (Figure 4D). The overlap coefficient was 1 for all the three cell types between the metabolites from differential metabolome analysis and the metabolites from the iMAT-based altered reactions. This suggests that the combined list of pathway terms associated with each of the differentially abundant metabolites (from the metabolome data) were included in the list of pathway terms associated with the metabolites observed in the iMAT-based altered reactions. The overlap coefficient was above 0.95 between the iMAT approach and the reporter metabolite approach. In contrast, overlap coefficients between metabolome analysis and the graph-based RMA were 0.89, 0.31, and 0.41 in neurons, astrocytes, and microglia, respectively. This observation suggests that while metabolome and reporter metabolite approaches capture metabolites with comparatively different functionalities, iMAT metabolites provide a broader list that covers both. Additionally, it is worth noting that metabolite abundance data typically exhibits considerably greater biological and technical variability compared to transcriptomic measurements.^69^ Therefore, the identification of consistent pathway-level perturbations across both omics layers, particularly under comparable statistical significance thresholds, underscores the robustness of the observed genotype-dependent metabolic alterations.

### Human postmortem brain transcriptome data validates key findings

To validate the findings from our study, an independent human transcriptome dataset^70^ was analyzed using the same pipeline. Mayo Clinic data were used to construct sample-based iMAT models. Significantly altered reactions between the *APOE3* and *APOE4* individuals were identified by Fisher’s exact test, and the metabolites of the altered reactions were subjected to pathway enrichment (*p* value < 0.05) (Figure S3A; Table S5). Only one of the terms enriched in Mayo clinic dataset did not appear in the iPSC dataset (Phenylalanine, tyrosine, and tryptophan biosynthesis). 99 pathways were common between at least one of the three iPSC-derived cell types and Mayo clinic results (super exact test *p* value = 1.73 × 10^−46^). These pathways include pathways of neurodegeneration, fatty acid metabolism, thyroid metabolism, biosynthesis of amino acids, amino acid metabolism, bile secretion, ABC transporters, and sphingolipid metabolism terms.

RMA was applied also to the Mayo clinic dataset. In the Mayo clinic results, 78 metabolites were identified as reporter metabolites. Then, metabolite-based pathway enrichment was performed similar to the iPSC data (Figure S3B; Table S5). As a result, 21 out of 27 terms linked to Mayo clinic pathways were common with iPSC results (super exact test *p* value = 1.21 × 10^−9^). These 21 pathways include pathways of neurodegeneration, fatty acid metabolism, glycerophospholipid metabolism, and purine and pyrimidine metabolism, thus providing a validation of the highest scored pathways of our results using an independent dataset.

## Discussion

In the present study, transcriptome data from iPSC-derived and *APOE3* or *APOE4* carrying neurons, astrocytes, and microglia were mapped to a human genome scale metabolic model to construct metabolic models by iMAT. Then, using these models, metabolic reactions with altered activities between *APOE3* and *APOE4* genotypes were determined. We also identified pathways overrepresented with the metabolites consumed/produced in the affected reactions. Our results show distinct pathways of lipid metabolism to be altered as consequences of the APOE4 allele. As an alternative transcriptomic approach, we used the graph-driven RMA method, which yielded complementary results by capturing metabolites and pathways both directly and indirectly related to lipid metabolism and energy metabolism. Finally, metabolome data from the same cell types highlighted differentially altered metabolites between *APOE3* and *APOE4* genotypes and associated pathways.

We also identified additional metabolic pathways associated with the *APOE4* genotype. This study shows that bile-acid metabolism may be related to *APOE* genotype and regulated differently in neurons and glia. Bile secretion is the most significant term in the reporter metabolite-based pathway enrichment of neurons in our study. In the metabolite-based pathway enrichment analysis, primary bile acid biosynthesis was common across all iMAT, reporter metabolite and metabolome-based analyses in neurons, and also across iMAT and metabolome-based analyses in astrocyte and microglia. For microglia, one of the five metabolites found to be differentially abundant based on the metabolome results was associated with bile acid biosynthesis (27-hydroxycholesterol). Another interesting finding from our analyses is cell-type specific differential thyroid hormone metabolism associated with the APOE4 variant. Thyroid hormone metabolism is of clinical relevance as abnormalities in thyroid function are established causes of treatable cognitive impairment.^57^ To the best of our knowledge, the association of intracellular thyroid metabolism with the *APOE* genotype in different brain cell types has not been previously reported. Metabolites related to thyroid hormone metabolism were identified as reporter metabolites in neurons and astrocytes. This metabolism was also enriched in all three cell types as well as in the validation dataset when metabolites of iMAT-based altered reactions were used. Since thyroid-related metabolites were not measured in the *APOE* metabolome data, these metabolites were not in the list of differentially abundant metabolites. Although they have previously been linked to dementia,^60^^,^^61^^,^^71^ this study provides a detailed analysis of their association with *APOE4* genotype through alterations in the intracellular pathway. Additionally, we showed that the *APOE4* variant affects lipid metabolism in neurons and glia while fatty acid oxidation was altered between *APOE3* versus *APOE4* variants in all cell types.

### Cholesterol biosynthesis

**Figure 5:**
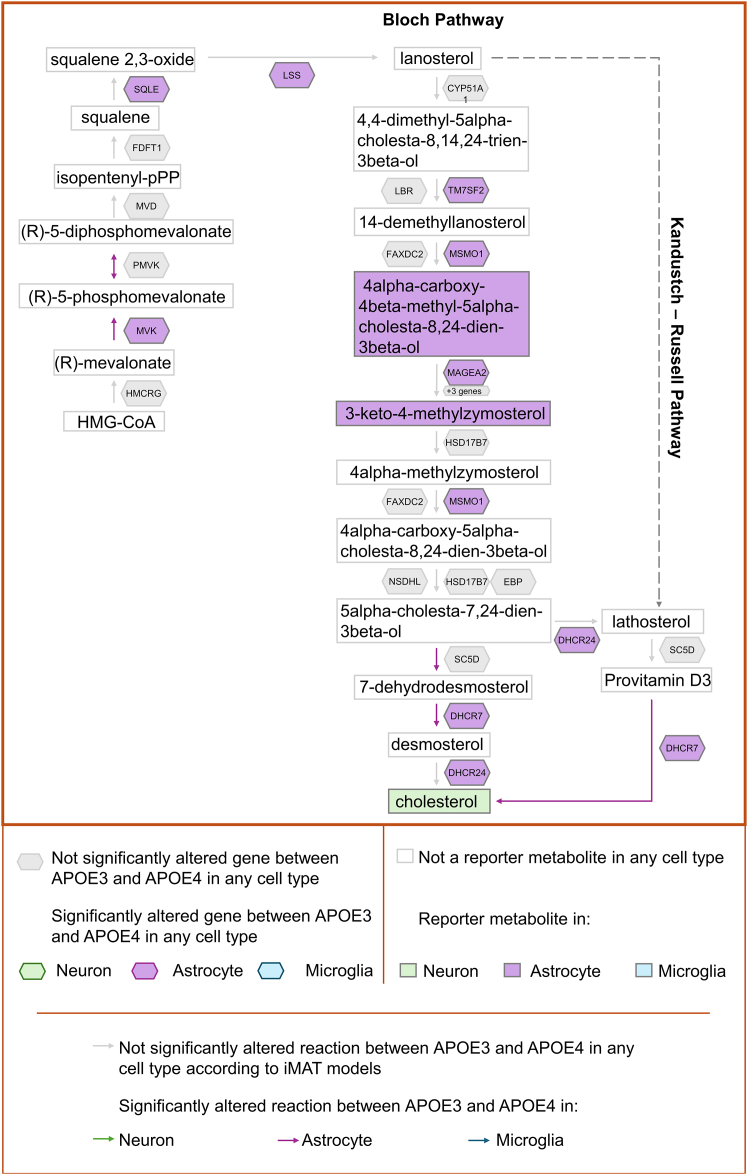
Cholesterol biosynthesis metabolism Human cholesterol biosynthesis metabolism (Bloch pathway) is illustrated. In the illustration, metabolites are represented by rectangles, genes by hexagons and reactions by arrows. Reactions, reporter metabolites and genes significantly altered between APOE3 and APOE4 metabolic models found in this study are colored for different cell types (green: neuron, purple: astrocyte and blue: microglia).

The integrated mapping of all three approaches highlights astrocyte-specific perturbations in the cholesterol biosynthesis pathway—a process long recognized for its strong association with APOE—as anticipated (Figure 5). Astrocytes are the main site of cholesterol synthesis and *APOE* expression in the brain.^72^ Our findings pinpoint alterations between *APOE3* and *APOE4* genotypes in the Bloch pathway arm of cholesterol biosynthesis (Figure 5), which is active in these cells.^73^ Specifically, iMAT identified five altered reactions within the astrocyte cholesterol pathway (detailed in Figure 5). Building on this, both 4alpha-carboxy-4beta-methyl-5alpha-cholesta-8,24-dien-3beta-ol and 3-keto-4-methylzymosterol were predicted as key reporter metabolites, further reinforcing the metabolic shift observed in astrocytes. Furthermore, several cholesterol biosynthesis genes (*MVK, SQLE, LSS,* *TM7SF2,* *MSMO1,* *MAGEA2,* *DHCR7,* and *DHCR24*) were differentially expressed in astrocytes, confirming that APOE genotype affects cholesterol synthesis in this cell type. In contrast, no alterations were found in the neuron-specific Kandutsch-Russell pathway, consistent with the observation that astrocytes are the main cholesterol source for adult neurons.^72^

**Figure 6:**
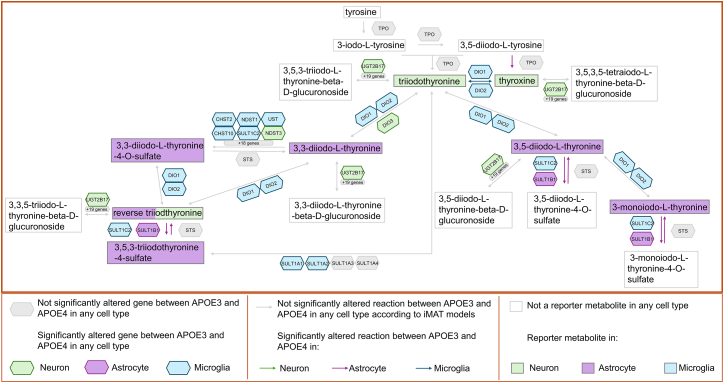
Thyroid hormone metabolism Human thyroid hormone metabolism is illustrated. In the illustration, metabolites are represented by rectangles, genes by hexagons and reactions by arrows. Altered reactions, reporter metabolites, and genes whose expression differs significantly between APOE3 and APOE4 are color-coded by cell type: green for neurons, purple for astrocytes, and blue for microglia. The drawing is based on cytoplasmic reactions, but the cellular compartmental difference was ignored while mapping the results of the study.

**Figure 7:**
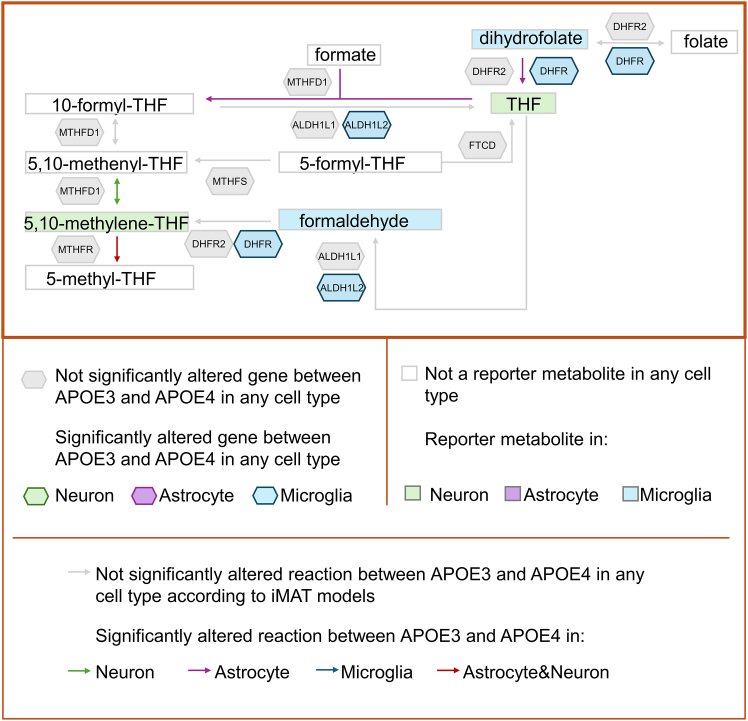
Folate metabolism Human folate metabolism is illustrated. In the illustration, metabolites are represented by rectangles, genes by hexagons and reactions by arrows. Altered reactions, reporter metabolites, and genes whose expression differs significantly between APOE3 and APOE4 are color-coded by cell type: green for neurons, purple for astrocytes, and blue for microglia. The drawing is based on cytoplasmic reactions, but the cellular compartmental difference was ignored while mapping the results of the study.

**Figure 8:**
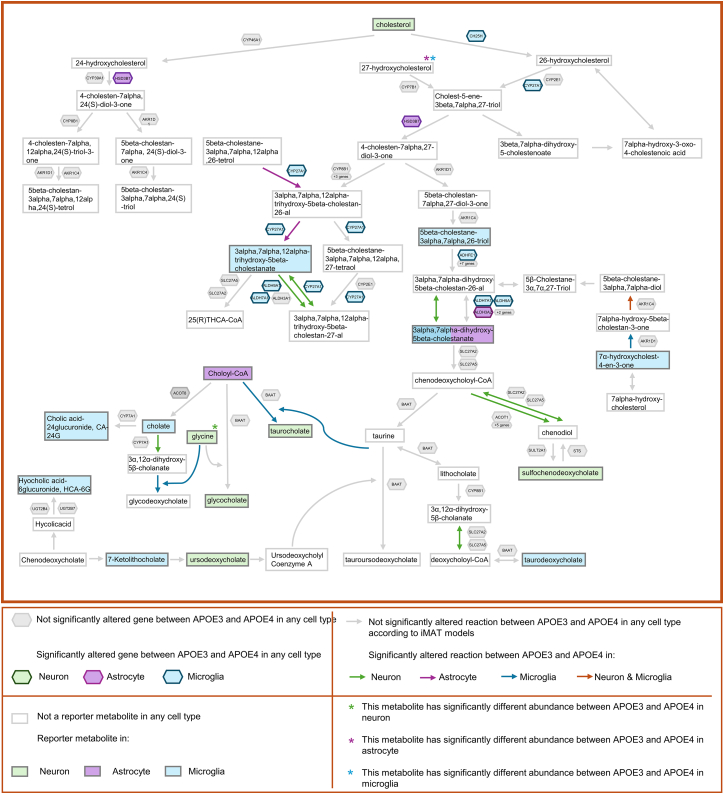
Bile acid biosynthesis metabolism Human bile acid biosynthesis is illustrated. In the illustration, metabolites are represented by rectangles, genes by hexagons and reactions by arrows. Altered reactions, reporter metabolites, and genes whose expression differs significantly between APOE3 and APOE4 are color-coded by cell type: green for neurons, purple for astrocytes, and blue for microglia. The drawing is based on cytoplasmic reactions, but the cellular compartmental difference was ignored while mapping the results of the study.

Encouraged by these findings, we present results for thyroid hormone metabolism, folate metabolism, and bile acid biosynthesis below, which were commonly identified to be altered in different cell types in our study. Their association with the *APOE* genotype and their mechanisms in different brain cell types have not been extensively studied in AD, and we provide valuable insights into these pathways in terms of the effect of *APOE* genotype. For this purpose, pathway maps for these three pathways were generated based on metabolic atlas database.^74^ On these maps, reporter metabolites, significantly affected reactions based on the iMAT models and differentially expressed genes were marked (Figures 6, 7, and 8).

### Thyroid hormone metabolism

While thyroid hormone metabolism has been linked to AD and dementia through extracellular hormone levels,^57^^,^^75^ intracellular expression of thyroid hormone genes in brain (e.g.,.*DIO2* and *DIO3*) is reported in human protein atlas.^76^ In line with this, we present evidence of intracellular alterations in this metabolism between *APOE3* and *APOE4* states in a cell type specific manner (Figure 6). In neurons, the key hormones triiodothyronine (T3) and thyroxine (T4) were identified as reporter metabolites. In astrocytes, metabolic products of these hormones were reporter metabolites. Interestingly, the reaction producing thyroxine from 3,5-diiodo-L-thyroxine was significantly altered in astrocyte iMAT models, despite no change in the controlling gene’s expression, suggesting post-transcriptional regulation. Reverse triiodothyronine (rT3), an inactivation product, was a reporter metabolite in both neurons and astrocytes, with associated reactions and genes being significantly altered in astrocytes. This aligns with findings that oxidative stress in AD can decrease the levels of T3 and increase rT3 levels.^75^

### Folate metabolism

Folate metabolism is a route through which precursor molecules are produced for the biosynthesis of nucleotides and the synthesis of various vitamins. It is also known that folate deficiency is a risk factor for AD.^77^ Our results show distinct patterns of APOE-related disruption in folate metabolism across different brain cell types (Figure 7). We found that dihydrofolate was a reporter metabolite in microglia, while its product, tetrahydrofolate (THF), was a reporter metabolite in neurons. Additionally, formaldehyde produced from THF was a reporter metabolite in microglia, and 5,10-methylene-THF was a reporter metabolite in neurons. In addition, reactions producing THF from dihydrofolate and producing 10-formyl-THF from THF were significantly altered in the iMAT models of astrocytes. The reduced activity of the 10-formyl-THF-producing reaction in APOE4 astrocyte models is consistent with reports of lower levels of this metabolite in the cerebrospinal fluid of AD patients.^78^ Interestingly, expression of MTHFD1, the gene controlling this reaction, was not significantly altered in any cell type. Another reaction also controlled by this gene (5,10-methenyl-THF <-> 5,10-methylene-THF) was found to be significantly altered in the iMAT models of neurons. Mutations in this gene were previously shown to be associated with AD and implicated in alterations of folate metabolism.^78^ Therefore, although no change was observed at the transcriptome level, iMAT-based integration of transcriptome data into metabolic models captured the reactions associated with the *MTHFD1* gene, highlighting the power of integrating transcriptomic data into metabolic models (Figure 7).

The identification of thyroid and folate metabolism as potential pathways in our analysis is intriguing; however, given that specific metabolites were not directly measured, these associations remain indirect. Recent experimental and proteogenomic studies provide context for these observations, suggesting that these pathways may be relevant to AD pathology. Specifically, Cook et al.^79^ demonstrated that L-thyroxine may be a key druggable target in AD by linking thyroid hormone signaling to specific risk genes through multi-omics analysis. Parallel to this, the relevance of folate metabolism is highlighted by its role in regulating homocysteine levels.^80^ Zhang et al.^81^ showed that selenium-folate interventions in AD models directly reduced amyloid beta and tau pathology while modulating lipid metabolism, including sphingolipids and fatty acids. Additionally, female *APOE4* homozygous AD patients show higher^82^ elevated homocysteine than *APOE4* non-carriers. While our current data do not provide direct validation, these studies collectively suggest that thyroid and folate pathways warrant further targeted investigation as metabolic modulators of AD pathogenesis, particularly in relation to *APOE*-mediated risk.

### Bile acid biosynthesis

The study also provides valuable insights into how *APOE* genotype affects bile acid biosynthesis (Figure 8), a pathway that begins with cholesterol.^42^ Cholesterol itself was identified as a reporter metabolite in neurons. In microglia, cholate, a primary bile acid linked to increased blood-brain barrier permeability in AD,^83^ was a reporter metabolite. Cholyl-CoA, used in the production of cholate, was identified as a reporter metabolite in astrocytes. The reaction metabolizing cholate to form glycodeoxycholate was also found to be significantly altered in the microglia iMAT models. Glycine, another substrate for this reaction, was found to be both a reporter metabolite in neurons and had a significantly altered abundance in the metabolome data. Several derivatives of the primary bile acid, chenodeoxycholate, including ursodeoxycholate in neurons,^84^ were identified as reporter metabolites. These findings reveal a distinct association between *APOE* genotype and specific reactions and metabolites in the bile acid pathway, offering valuable insights to pathogenic mechanisms in AD.

In summary, Figures 5, 6, 7, and 8 illustrate how the different analyses performed in this study complement each other. Furthermore, a comprehensive metabolite-centric approach is presented in terms of providing cell type-specific and high-resolution results. In conclusion, we have demonstrated the integration of transcriptomic and metabolic data via different bioinformatics methods that yield both confirmatory and valuable insights into cell type specific biology of *APOE* alleles. The information extracted from the metabolome data showed a high overlap with the pathways obtained by mapping transcriptome data on a human genome-scale metabolic network by iMAT. In this work, we present a powerful framework for the metabolite-centric integration of transcriptomic and metabolomic data with GEMs. This framework offers a comprehensive approach, emphasizing the central role of specific metabolites and providing pathway-level outputs. Our results on *APOE* genotypes and pathway associations not only validate the presented framework but are also consistent with previous results in the literature, underscoring the relevance of our approach. These findings are expected to guide subsequent integrated analysis of multi-omics data to elucidate complex behavior of cells and enhance our understanding of the role of *APOE* in Alzheimer’s disease.

### Limitations of the study

Although this study provides valuable and important insights into the metabolic effects of the *APOE* genotype, acknowledging the limitations of the current experimental model is essential for the proper interpretation of our findings. The primary limitation comes from the low sample size across all experimental groups in this iPSC-derived model system. The limited sample size meant that formal statistical tests could not be appropriately applied to compare sample-based metabolic models of *APOE3* and *APOE4*. Furthermore, it reduced the statistical power for the metabolome data analysis. Yet, using the nominal *p* values maximized the capture of metabolites with altered abundance, which served as input for pathway analysis, despite the known statistical limitation. It also enabled the identification of pathways known to be relevant in AD. Similarly, enrichment results were reported using nominal *p* values to preserve biological associations, corresponding to FDR values of up to 0.1. This FDR threshold, together with the limited number of resulting enriched terms, helps limit false discovery risk. This approach facilitates the exploratory analysis of metabolic pathways associated with *APOE*-genotype. Additionally, the isogenic background of iPSC-derived cells used in this study precluded inclusion of covariates like age, sex, and AD neuropathology in our analyses. Future research should include both male and female backgrounds to explore the known interactions between sex and the *APOE4* genotype in AD. Finally, validating thyroid and folate metabolism through targeted assays and *APOE4*-specific models represents an important direction for future studies. In addition, integration of fluxomics or stable isotope tracing could provide additional support for transcriptome-based predictions and establish mechanistic links between metabolic changes and specific neuronal or glial phenotype.

## Resource availability

### Lead contact

Requests for further information and resources should be directed to the lead contact, Madhav Thambisetty (thambisettym@mail.nih.gov).

### Materials availability

This study did not generate new unique reagents.

### Data and code availability

• Deposited Data: Raw sequencing data of microglia samples and processed read counts have been deposited at GEO: GSE305481) and are publicly available. Previously published iPSC data are available under accession GEO: GSE102956.
• Software and Custom Code: All original code for the iMAT and Reporter Metabolite analyses has been deposited at Zenodo and is publicly available at Zenodo Data: https://doi.org/10.5281/zenodo.16037670. The code includes custom scripts for multi-omics integration and visualization.
• Any additional information required to reanalyze the data reported in this paper is available from the lead contact upon request.

## Acknowledgments

This work was supported in part by the intramural programs of the 10.13039/100000049National Institute on Aging, the 10.13039/100000062National Institute of Diabetes and Digestive and Kidney Diseases, and the 10.13039/100000065National Institute of Neurological Disorders and Stroke within the 10.13039/100000002National Institutes of Health. This research was also enabled by a Collaborative Translational Initiative grant from 10.13039/100000065NINDS. The contributions of the NIH author(s) are considered Works of the United States Government. The findings and conclusions presented in this paper are those of the author(s) and do not necessarily reflect the views of the NIH or the U.S. Department of Health and Human Services. Dilara Uzuner Odongo is funded by 10.13039/501100004410TUBITAK 2211-A PhD Student Scholarship and 10.13039/501100007246Council of Higher Education 100/2000 PhD Scholarship Programmes.

## Author contributions

Conceptualization, D.U.O., P.S.N., T.Ç., and M.T.; methodology, D.U.O., P.S.N., T.Ç., and M.T.; software, D.U.O.; formal analysis, D.U.O.; investigation, D.U.O., R.A.S., L.C., and L.G.Y.; data curation, D.U.O.; writing – original draft, D.U.O.; writing – review and editing, D.U.O., P.S.N., T.Ç., and M.T.; visualization, D.U.O.; supervision, P.S.N., T.Ç., and M.T.

## Declaration of interests

The authors declare no competing interests.

## Declaration of generative AI and AI-assisted technologies in the writing process

During the preparation of this work the author(s) used Google’s Gemini in order to improve the readability and language of the manuscript. After using this tool/service, the author(s) reviewed and edited the content as needed and take(s) full responsibility for the content of the publication.

## STAR★Methods

### Key resources table

**Table:** 

REAGENT or RESOURCE	SOURCE	IDENTIFIER
**Antibodies**		

Anti-Neurofilament Marker (pan-neuronal, cocktail)	BioLegend	BioLegend Cat# 837802; RRID:AB_2565384
Purified anti-Tubulin β-3 (TUBB3)	BioLegend	BioLegend Cat# 802001; RRID:AB_2564645
Anti-S100B Antibody mouse monoclonal, SH-B1	Sigma Aldrich	Sigma-Aldrich Cat# S2532; RRID:AB_477499
Chicken Anti-Glial Fibrillary Acidic Protein (GFAP) Polyclonal antibody, Unconjugated	Millipore Sigma	Millipore Cat# AB5541; RRID:AB_177521
Anti-Iba1	Novus Biologicals	Novus Cat# NB100-1028; RRID:AB_3148646
Anti-CX3CR1	Abcam	Abcam Cat #ab8020; RRID:AB_306202

**Chemicals, peptides, and recombinant proteins**		

Recombinant Human M-CSF	PeproTech	Cat#300-03
Recombinant Human M-CSF	PeproTech	Cat#300-25
Recombinant Human IL-34	PeproTech	Cat#200-34
Recombinant Human TGF-β1 (HEK293 derived)	PeproTech	Cat#100-21
Recombinant Human Fractalkine (CX3CL1)	PeproTech	Cat#300-31
Human BMP-4	PeproTech	Cat#120-05
Human FGF-basic	PeproTech	Cat# 100-18c

**Deposited data**		

Raw counts for RNA-seq (neuron and astrocytes)	Lin et al.^20^	GEO: GSE102956
Raw counts for RNA-seq (microglia)	This paper	GEO: GSE305481
Mayo Clinic RNAseq data	Synapse	https://www.synapse.org/Synapse:syn5550404

**Experimental models: Cell lines**		

Human: isogenic *APOE3/APOE3* and *APOE4/APOE4* iPSCs from parental line Coriell #AG09173	Obtained via MTA from MIT	Coriell #AG09173; RRID: CVCL_4L66

**Software and algorithms**		

Gurobi v.8.0.1	Gurobi	https://www.gurobi.com/
MATLAB 2020a	Mathworks	https://www.mathworks.com/
MBROLE 3.0	López-Ibáñez et al.^85^	https://doi.org/10.1093/nar/gkad405
iMAT	Zur et al.^9^	https://doi.org/10.1093/bioinformatics/btq602
COBRA Toolbox	Heirendt et al.^86^	https://doi.org/10.1038/s41596-018-0098-2
Cytoscape 3.10.3	Cytoscape	https://cytoscape.org/
Fiji	ImageJ	https://doi.org/10.1038/nmeth.2019

**Other**		

Codes for model generation and simulation	GitHub	https://github.com/SysBioGTU/MetaboliteCentricGSMM
Codes for model generation and simulation	Zenodo	https://doi.org/10.5281/zenodo.16037670
STEMDIFF™ HEMATOPOIETIC KIT	Stem Cell Technologies	Cat#05310
STEMDIFF™ MICROGLIA DIFFERENTIATION KIT	Stem Cell Technologies	Cat#100-0019
STEMDIFF™ MICROGLIA MATURATION KIT	Stem Cell Technologies	Cat#100-0020
Hoechst 33342, Trihydrochloride, Trihydrate	ThermoFisher Scientific	ThermoFisher Cat# H3570

### Experimental model and study participant details

#### Human induced pluripotent stem cells

This study utilized an isogenic set of human induced pluripotent stem cells (iPSCs) to derive microglia. The parental line (Coriell# AG09173), originally from a healthy individual, was edited via CRISPR-Cas9 to generate APOE3 and APOE4 carrying lines. The isogenic background used in this study (AG09173) is derived from a female (age 75) donor. Due to the isogenic design, sex-specific differences could not be evaluated, which is detailed as a study limitation in the discussion section. All iPSC differentiation and data analysis procedures were performed in accordance with the guidelines and oversight of the Intramural Research Program of the National Institute on Aging (NIA) and the National Institutes of Health (NIH).

### Method details

#### iPSC maintenance and derivation

Prior to differentiation, all iPSC derived cells were maintained in mTeSR Plus media (StemCell) and confirmed to be karyotypically normal. iPSCs were differentiated into microglia using protocol previously described.^20^^,^^87^ Following differentiation, cells were stained for canonical cell type-specific markers (Figure S1). 1 million cells were harvested per replicate across at least two independent derivations.

### Quantification and statistical analysis

#### Transcriptome dataset

Transcriptome data generated by Lin et al.^20^ was downloaded from the GEO database^88^ in the form of raw counts (GEO accession code: GSE102956). In this previously published work, *APOE3*-expressing induced pluripotent stem cells (iPSCs, Coriell# AG09173, female age 75) taken from a healthy individual were edited to generate isogenic, *APOE4*-expressing cells using the CRISPR-Cas9 genome editing. Then, *APOE3* and *APOE4* carrying isogenic iPSC lines were differentiated into neurons, astrocytes, and microglia-like cells. RNA sequencing was performed on all three cell types homozygous for either the *APOE3* or *APOE4* allele.^20^ Samples were allocated to experimental groups based on their APOE genotype (*APOE3* vs. *APOE4*). Neuron and astrocyte cells have three replicates for both *APOE3* and *APOE4* groups. Since the publication of these datasets, microglia derivation protocols have been further optimized. We therefore chose to generate our own microglial transcriptomic data from the same iPS cells (GEO accession code: GSE305481) derived using a well-established protocol.^87^ Raw sequencing data of microglia samples in the form of .fastq files were processed with the same pipeline described in Lin et al.,^20^ and raw read counts were generated. This data also includes three replicates for both *APOE3* and *APOE4* groups. geTMM normalization^89^ was applied on the raw counts for iMAT-based metabolic network integration after the genes with very low or zero expression values in at least half of the samples (counts per million <0.1) were removed. Differential gene expression analysis between APOE3 and APOE4 samples was performed using DeSeq2 by using raw counts.^90^ The gene symbols of transcriptome data were converted to Ensembl Gene IDs to be compatible with the metabolic model, Human-GEM (see below).

The Mayo Clinic^70^ transcriptome data, which was previously processed from raw RNA-seq sequencing data to raw read counts in another study,^91^ was used as a validation dataset. The effect of age, sex, post-mortem interval and disease covariates in the data was adjusted. 81 APOE3 and 33 APOE4 samples in the dataset were included in the analysis. Similar to above, geTMM normalization was applied to the raw read counts prior to the iMAT analysis. Differential gene expression analysis between *APOE3* and *APOE4* samples was performed using DeSeq2 by using raw counts.

#### Metabolome dataset

Metabolome data includes *APOE3* and *APOE4* expressing neuron, astrocyte and microglia cells. There are 4 samples for both *APOE3* and *APOE4* expressing neurons, 7 samples for *APOE3* microglia, 6 samples for *APOE4* microglia, 6 samples for *APOE3* astrocytes and 4 samples for *APOE4* astrocytes. 499 metabolites were measured for each sample (Table S6). Measurements were derived from 5 different assays: Oxysterol assay, Biocrates AbsoluteIDQ® p180, Biocrates AbsoluteIDQ® Bile acids, Fatty acid assay (free), and Lipid assay. Metabolites with NA values in more than 30% of the samples were removed from the data for each cell type separately. Other NA readings were assigned with the half of the limit of detection value of each assay. Accordingly, the remaining number of metabolites were 245, 229 and 210 for neurons, astrocytes, and microglia, respectively. Student’s t-test was applied to the log2-transformed data to identify metabolites with significantly different abundance between *APOE3* and *APOE4* states for each cell type.

#### iMAT analysis

Integrative Metabolic Analysis Tool (iMAT)^9^ was used to reconstruct metabolic models for each sample by mapping the transcriptome data on Human-GEM v.1.10,^21^ which includes 13,078 reactions associated with 3,625 genes. The iMAT algorithm aims to predict inactive reactions in a GEM in each condition by using transcriptome data. To this end, the algorithm uses the list of highly and lowly expressed genes, determined based on user-defined thresholds, and categorizes the reactions of the GEM as active or inactive by solving an optimization problem constrained by the mass-balances around intracellular metabolites. The genes with expression values below the first quartile of averaged expression values of a given cell type were considered as lowly expressed in the iMAT analysis while the genes with expression values greater than the third quartile were considered as highly expressed.^27^ geTMM normalized counts^89^ were used to identify the thresholds.^92^ Gurobi v.8.0.1 optimization tool was used to solve mixed-integer linear programming-based optimization problems within the iMAT algorithm. After iMAT predicts the active and inactive reactions for each sample, models were binarized by representing inactive reactions as 0 and active reactions as 1. iMAT simulations were performed using the built-in function in the COBRA Toolbox^86^ under MATLAB 2020a environment. Principal Component Analysis was performed on the binarized models using the “logisticSVD” function of logisticPCA R package.^93^

To determine the reactions that were activated/inactivated between the *APOE3* and *APOE4* states in each cell type, the number of samples in which they were active were compared between the two states. Because of the small sample size, the altered reactions were chosen as follows: (i) The reactions that are fully active in one *APOE* status and fully inactive in the other *APOE* status were considered to be altered. (ii) Also, the reactions active (inactive) in all *APOE3* samples and inactive (active) in only one *APOE4* sample (designated as 3-1 case) were also considered to be altered. (iii) Similarly, reactions active (inactive) in two *APOE3* samples and inactive (active) in all *APOE4* samples (designated as 2-0 case) were also considered to be altered. To evaluate the sensitivity and statistical significance of the alteration criteria, we performed an exact permutation test and a Monte Carlo random sampling analysis. The detailed methodology and thresholds used for these validations are provided in the Technical Note S1. Significance of overlap between the altered reactions and associated metabolites of different cell types was calculated by Super Exact Test.^94^

#### Reporter metabolite analysis

First, a bipartite gene-metabolite interaction graph was constructed from Human-GEM. In this graph, metabolites and genes are nodes, and an edge between a metabolite and a gene represents their involvement in the same reaction. The graph included 3,625 genes and 6,888 metabolites. To map transcriptome data to the constructed gene-metabolite interaction network, p-values of the genes obtained from the differential gene expression analysis were used. Metabolites in the network were then scored by using the averages of z- transformed p-values of neighbour genes,^22^ which correspond to the genes of reactions that use the metabolite of interest as substrate or product. Then, these scores were converted to p-values for each metabolite. Metabolites with significant p-values (p-value < 0.05) were defined as reporter metabolites.

All calculations were performed in MATLAB R2020a using in-house codes.

#### Pathway enrichment analysis for metabolites

Pathway enrichment analysis was applied to the metabolites for three different analyses by using MBROLE 3.0^85^: (i) the list of metabolites with significantly altered abundance based on the metabolome data, (ii) the reporter metabolites, (iii) the metabolites taking place in the reactions determined to be altered by the iMAT approach. Enrichment analysis was performed separately for each cell type. KEGG pathway annotations were retrieved with *Homo sapiens* background set. The terms with a p-value less than 0.05 were selected as significant.

As an alternative approach for the pathway-based comparison of identified metabolites by three different analyses, the pathway annotations for each metabolite were downloaded from the MBROLE database. Metabolites identified by different analyses (differential metabolome, reporter metabolites and metabolites from the altered reactions) were compared in terms of the similarity of their pathway annotations. The overlap coefficient (OC) was used to determine the similarity between the annotation lists. The OC is a modified version of the Jaccard index.^95^ Here, given two metabolite annotation lists from two different analyses, M_1_ and M_2_, with associated annotations A_1_ and A_2_, OC is computed using the formula below:

$$OC\left(M_{1},M_{2}\right)=\frac{\left|A_{1}\cap A_{2}\right|}{\min\left(\left|A_{1}\right|,\left|A_{2}\right|\right)}$$

## Supplemental information

Document S1. Figures S1–S3 and Technical Note S1

Data S1. Cytoscape session file for the network in Figure 2C

Table S1. iMAT-based perturbed reactions in each cell type, related to Figure 2

Table S2. Metabolite-based pathway enrichment analysis of iPSCs, related to Figures 2 and 4

Table S3. Reporter Metabolite Analysis Results of iPSCs, related to Figure 3

Table S4. Significantly changed metabolites of iPSCs metabolome data

Table S5. Metabolite-based pathway enrichment analysis of Mayo Clinic

Table S6. Metabolome Data Abundances, related to STAR Methods

## References

[1] Mardinoglu A., Gatto F., Nielsen J. (2013). Genome-scale modeling of human metabolism - a systems biology approach. Biotechnol. J..

[2] Chowdhury S., Fong S.S. (2020). Leveraging genome-scale metabolic models for human health applications. Curr. Opin. Biotechnol..

[3] Agren R., Bordel S., Mardinoglu A., Pornputtapong N., Nookaew I., Nielsen J. (2012). Reconstruction of Genome-Scale Active Metabolic Networks for 69 Human Cell Types and 16 Cancer Types Using INIT. PLoS Comput. Biol..

[4] Wang Y., Eddy J.A., Price N.D. (2012). Reconstruction of genome-scale metabolic models for 126 human tissues using mCADRE. BMC Syst. Biol..

[5] Stempler S., Yizhak K., Ruppin E. (2014). Integrating transcriptomics with metabolic modeling predicts biomarkers and drug targets for Alzheimer's disease. PLoS One.

[6] Abdik E., Çakır T. (2021). Systematic investigation of mouse models of Parkinson's disease by transcriptome mapping on a brain-specific genome-scale metabolic network. Mol. Omics.

[7] Folger O., Jerby L., Frezza C., Gottlieb E., Ruppin E., Shlomi T. (2011). Predicting selective drug targets in cancer through metabolic networks. Mol. Syst. Biol..

[8] Valcárcel L.V., Torrano V., Tobalina L., Carracedo A., Planes F.J. (2019). rMTA: robust metabolic transformation analysis. Bioinformatics.

[9] Zur H., Ruppin E., Shlomi T. (2010). iMAT: an integrative metabolic analysis tool. Bioinformatics.

[10] Lee S., Zhang C., Kilicarslan M., Piening B.D., Bjornson E., Hallström B.M., Groen A.K., Ferrannini E., Laakso M., Snyder M. (2016). Integrated Network Analysis Reveals an Association between Plasma Mannose Levels and Insulin Resistance. Cell Metab..

[11] Mardinoglu A., Agren R., Kampf C., Asplund A., Uhlen M., Nielsen J. (2014). Genome-scale metabolic modelling of hepatocytes reveals serine deficiency in patients with non-alcoholic fatty liver disease. Nat. Commun..

[12] Zelezniak A., Pers T.H., Soares S., Patti M.E., Patil K.R. (2010). Metabolic Network Topology Reveals Transcriptional Regulatory Signatures of Type 2 Diabetes. PLoS Comput. Biol..

[13] Çakır T. (2015). Reporter pathway analysis from transcriptome data: Metabolite-centric versus Reaction-centric approach. Sci. Rep..

[14] Liu C.C., Liu C.C., Kanekiyo T., Xu H., Bu G. (2013). Apolipoprotein E and Alzheimer disease: risk, mechanisms and therapy. Nat. Rev. Neurol..

[15] Fernandez C.G., Hamby M.E., McReynolds M.L., Ray W.J. (2019). The Role of APOE4 in Disrupting the Homeostatic Functions of Astrocytes and Microglia in Aging and Alzheimer's Disease. Front. Aging Neurosci..

[16] Yang L.G., March Z.M., Stephenson R.A., Narayan P.S. (2023). Apolipoprotein E in lipid metabolism and neurodegenerative disease. Trends Endocrinol. Metab..

[17] Kuehn B.M. (2020). In Alzheimer Research, Glucose Metabolism Moves to Center Stage. JAMA.

[18] Perry G., Nunomura A., Raina A.K., Aliev G., Siedlak S.L., Harris P.L.R., Casadesus G., Petersen R.B., Bligh-Glover W., Balraj E. (2003). A Metabolic Basis for Alzheimer Disease. Neurochem. Res..

[19] Mahajan U.V., Varma V.R., Griswold M.E., Blackshear C.T., An Y., Oommen A.M., Varma S., Troncoso J.C., Pletnikova O., O'Brien R. (2020). Dysregulation of multiple metabolic networks related to brain transmethylation and polyamine pathways in Alzheimer disease: A targeted metabolomic and transcriptomic study. PLoS Med..

[20] Lin Y.T., Seo J., Gao F., Feldman H.M., Wen H.L., Penney J., Cam H.P., Gjoneska E., Raja W.K., Cheng J. (2018). APOE4 Causes Widespread Molecular and Cellular Alterations Associated with Alzheimer's Disease Phenotypes in Human iPSC-Derived Brain Cell Types. Neuron.

[21] Robinson J.L., Kocabaş P., Wang H., Cholley P.-E., Cook D., Nilsson A., Anton M., Ferreira R., Domenzain I., Billa V. (2020). An atlas of human metabolism. Science Signal..

[22] Patil K.R., Nielsen J. (2005). Uncovering transcriptional regulation of metabolism by using metabolic network topology. Proc Natl Acad Sci USA.

[23] Feringa F.M., Koppes-den Hertog S.J., Wang L.Y., Derks R.J.E., Kruijff I., Erlebach L., Heijneman J., Miramontes R., Pömpner N., Blomberg N. (2025). The Neurolipid Atlas: a lipidomics resource for neurodegenerative diseases. Nat. Metab..

[24] Styr B., Gonen N., Zarhin D., Ruggiero A., Atsmon R., Gazit N., Braun G., Frere S., Vertkin I., Shapira I. (2019). Mitochondrial Regulation of the Hippocampal Firing Rate Set Point and Seizure Susceptibility. Neuron.

[25] Moolamalla S.T.R., Vinod P.K. (2020). Genome-scale metabolic modelling predicts biomarkers and therapeutic targets for neuropsychiatric disorders. Comput. Biol. Med..

[26] Kutay M., Gozuacik D., Çakır T. (2022). Cancer Recurrence and Omics: Metabolic Signatures of Cancer Dormancy Revealed by Transcriptome Mapping of Genome-Scale Networks. OMICS A J. Integr. Biol..

[27] Lüleci H.B., Uzuner D., Çakır T., Thambisetty M., Chun J. (2023). Alzheimer’s Disease: Methods and Protocols.

[28] Rapp A., Gmeiner B., Hüttinger M. (2006). Implication of apoE isoforms in cholesterol metabolism by primary rat hippocampal neurons and astrocytes. Biochimie.

[29] Blumenfeld J., Yip O., Kim M.J., Huang Y. (2024). Cell type-specific roles of APOE4 in Alzheimer disease. Nat. Rev. Neurosci..

[30] Bandaru V.V.R., Troncoso J., Wheeler D., Pletnikova O., Wang J., Conant K., Haughey N.J. (2009). ApoE4 disrupts sterol and sphingolipid metabolism in Alzheimer's but not normal brain. Neurobiol. Aging.

[31] Snowden S.G., Ebshiana A.A., Hye A., An Y., Pletnikova O., O’Brien R., Troncoso J., Legido-Quigley C., Thambisetty M. (2017). Association between fatty acid metabolism in the brain and Alzheimer disease neuropathology and cognitive performance: A nontargeted metabolomic study. PLoS Med..

[32] Sienski G., Narayan P., Bonner J.M., Kory N., Boland S., Arczewska A.A., Ralvenius W.T., Akay L., Lockshin E., He L. (2021). APOE4 disrupts intracellular lipid homeostasis in human iPSC-derived glia. Sci. Transl. Med..

[33] Qi G., Mi Y., Shi X., Gu H., Brinton R.D., Yin F. (2021). ApoE4 Impairs Neuron-Astrocyte Coupling of Fatty Acid Metabolism. Cell Rep..

[34] Ioannou M.S., Jackson J., Sheu S.H., Chang C.L., Weigel A.V., Liu H., Pasolli H.A., Xu C.S., Pang S., Matthies D. (2019). Neuron-Astrocyte Metabolic Coupling Protects against Activity-Induced Fatty Acid Toxicity. Cell.

[35] Garcia Corrales A.V., Haidar M., Bogie J.F.J., Hendriks J.J.A. (2021). Fatty Acid Synthesis in Glial Cells of the CNS. Int. J. Mol. Sci..

[36] Goulding D.S., Williams H.C., Gorman A.A., Devanney N.A., Harrison D.A., Walsh A.E., Tuck T., Zajac D.J., Macauley S.L., Estus S. (2026). APOE4 drives maladaptive heterogeneity and immunometabolic responses of astrocytes. J. Neuroinflammation.

[37] Farmer B.C., Walsh A.E., Kluemper J.C., Johnson L.A. (2020). Lipid Droplets in Neurodegenerative Disorders. Front. Neurosci..

[38] Yin F. (2023). Lipid metabolism and Alzheimer's disease: clinical evidence, mechanistic link and therapeutic promise. FEBS J..

[39] Stephenson R.A., Sepulveda J., Johnson K.R., Lita A., Gopalakrishnan J., Acri D.J., Beilina A., Cheng L., Yang L.G., Root J.T. (2025). Triglyceride metabolism controls inflammation and microglial phenotypes associated with APOE4. Cell Rep..

[40] Haney M.S., Pálovics R., Munson C.N., Long C., Johansson P.K., Yip O., Dong W., Rawat E., West E., Schlachetzki J.C.M. (2024). APOE4/4 is linked to damaging lipid droplets in Alzheimer’s disease microglia. Nature.

[41] Guo J.L., Braun D., Fitzgerald G.A., Hsieh Y.T., Rougé L., Litvinchuk A., Steffek M., Propson N.E., Heffner C.M., Discenza C. (2025). Decreased lipidated ApoE-receptor interactions confer protection against pathogenicity of ApoE and its lipid cargoes in lysosomes. Cell.

[42] Baloni P., Funk C.C., Yan J., Yurkovich J.T., Kueider-Paisley A., Nho K., Heinken A., Jia W., Mahmoudiandehkordi S., Louie G. (2020). Metabolic Network Analysis Reveals Altered Bile Acid Synthesis and Metabolism in Alzheimer's Disease. Cell Rep. Med..

[43] Varma V.R., Wang Y., An Y., Varma S., Bilgel M., Doshi J., Legido-Quigley C., Delgado J.C., Oommen A.M., Roberts J.A. (2021). Bile acid synthesis, modulation, and dementia: A metabolomic, transcriptomic, and pharmacoepidemiologic study. PLoS Med..

[44] Fekkes D., van der Cammen T.J., van Loon C.P., Verschoor C., van Harskamp F., de Koning I., Schudel W.J., Pepplinkhuizen L. (1998). Abnormal amino acid metabolism in patients with early stage Alzheimer dementia. J. Neural Transm..

[45] Gueli M.C., Taibi G. (2013). Alzheimer’s disease: amino acid levels and brain metabolic status. Neurol. Sci..

[46] Liu P., Fleete M.S., Jing Y., Collie N.D., Curtis M.A., Waldvogel H.J., Faull R.L.M., Abraham W.C., Zhang H. (2014). Altered arginine metabolism in Alzheimer's disease brains. Neurobiol. Aging.

[47] Piubelli L., Murtas G., Rabattoni V., Pollegioni L. (2021). The Role of D-Amino Acids in Alzheimer’s Disease. J. Alzheimers Dis..

[48] Le Douce J., Maugard M., Veran J., Matos M., Jégo P., Vigneron P.-A., Faivre E., Toussay X., Vandenberghe M., Balbastre Y. (2020). Impairment of Glycolysis-Derived l-Serine Production in Astrocytes Contributes to Cognitive Deficits in Alzheimer’s Disease. Cell Metab..

[49] Dejakaisaya H., Harutyunyan A., Kwan P., Jones N.C. (2021). Altered metabolic pathways in a transgenic mouse model suggest mechanistic role of amyloid precursor protein overexpression in Alzheimer’s disease. Metabolomics.

[50] Sertbaş M., Ülgen K., Cakır T. (2014). Systematic analysis of transcription-level effects of neurodegenerative diseases on human brain metabolism by a newly reconstructed brain-specific metabolic network. FEBS Open Bio.

[51] Yan X., Hu Y., Wang B., Wang S., Zhang X. (2020). Metabolic Dysregulation Contributes to the Progression of Alzheimer's Disease. Front. Neurosci..

[52] Lee S., Devanney N.A., Golden L.R., Smith C.T., Schwartz J.L., Walsh A.E., Clarke H.A., Goulding D.S., Allenger E.J., Morillo-Segovia G. (2023). APOE modulates microglial immunometabolism in response to age, amyloid pathology, and inflammatory challenge. Cell Rep..

[53] Yassine H.N., Self W., Kerman B.E., Santoni G., Navalpur Shanmugam N., Abdullah L., Golden L.R., Fonteh A.N., Harrington M.G., Gräff J. (2023). Nutritional metabolism and cerebral bioenergetics in Alzheimer's disease and related dementias. Alzheimer's Dement..

[54] Farmer B.C., Williams H.C., Devanney N.A., Piron M.A., Nation G.K., Carter D.J., Walsh A.E., Khanal R., Young L.E.A., Kluemper J.C. (2021). APOΕ4 lowers energy expenditure in females and impairs glucose oxidation by increasing flux through aerobic glycolysis. Mol. Neurodegener..

[55] Anat R., Arad K., Yair G., Abraham R., Stephen Z.L. (2022). Serum folate deficiency and the risks of dementia and all-cause mortality: a national study of old age. Evid. Base Ment. Health.

[56] Reynolds E.H. (2002). Folic acid, ageing, depression, and dementia. BMJ.

[57] Eslami-Amirabadi M., Sajjadi S.A. (2021). The relation between thyroid dysregulation and impaired cognition/behaviour: An integrative review. J. Neuroendocrinol..

[58] Cummings J.L. (1983). Treatable dementias. Adv. Neurol..

[59] Lai F., Mercaldo N.D., Wang C.M., Hersch M.S., Hersch G.G., Rosas H.D. (2021). Association between Hypothyroidism Onset and Alzheimer Disease Onset in Adults with Down Syndrome. Brain Sci..

[60] Marouli E., Yusuf L., Kjaergaard A.D., Omar R., Kuś A., Babajide O., Sterenborg R., Åsvold B.O., Burgess S., Ellervik C. (2021). Thyroid Function and the Risk of Alzheimer's Disease: A Mendelian Randomization Study. Thyroid.

[61] Salehipour A., Dolatshahi M., Haghshomar M., Amin J. (2023). The Role of Thyroid Dysfunction in Alzheimer's Disease: A Systematic Review and Meta-Analysis. J. Prev. Alzheimers Dis..

[62] Toraih E.A., Siddiqui S., Siddiqui S., Shirini K., Elfezzani N., Abdelmaksoud A., Elshazli R.M., Hussein M.H., Elmorsy E.M., Fawzy M.S. (2025). Thyroid Disorders as a Risk Factor for Neurodegenerative Proteinopathies: A Large-Scale Propensity Score-Matched Analysis. Neuroepidemiology.

[63] Schroeder A.C., Privalsky M.L. (2014). Thyroid hormones, t3 and t4, in the brain. Front. Endocrinol..

[64] Quinlan P., Horvath A., Eckerström C., Wallin A., Svensson J. (2020). Altered thyroid hormone profile in patients with Alzheimer’s disease. Psychoneuroendocrinology.

[65] Morte B., Bernal J. (2014). Thyroid Hormone Action: Astrocyte–Neuron Communication. Front. Endocrinol..

[66] Fonteh A.N., Harrington R.J., Tsai A., Liao P., Harrington M.G. (2007). Free amino acid and dipeptide changes in the body fluids from Alzheimer's disease subjects. Amino Acids.

[67] Jia J., Yin J., Zhang Y., Xu G., Wang M., Jiang H., Li L., Zeng X., Zhu D. (2023). Thioredoxin-1 Promotes Mitochondrial Biogenesis Through Regulating AMPK/Sirt1/PGC1α Pathway in Alzheimer's Disease. ASN Neuro.

[68] Frontiñán-Rubio J., Rabanal-Ruiz Y., Durán-Prado M., Alcain F.J. (2021). The Protective Effect of Ubiquinone against the Amyloid Peptide in Endothelial Cells Is Isoprenoid Chain Length-Dependent. Antioxidants.

[69] Lai W.Y., Otte K.A., Schlötterer C. (2023). Evolution of Metabolome and Transcriptome Supports a Hierarchical Organization of Adaptive Traits. Genome Biol. Evol..

[70] Allen M., Carrasquillo M.M., Funk C., Heavner B.D., Zou F., Younkin C.S., Burgess J.D., Chai H.S., Crook J., Eddy J.A. (2016). Human whole genome genotype and transcriptome data for Alzheimer's and other neurodegenerative diseases. Sci. Data.

[71] Schroeder A.C., Privalsky M.L. (2014). Thyroid Hormones, T3 and T4, in the Brain. Front. Endocrinol..

[72] Staurenghi E., Leoni V., Lo Iacono M., Sottero B., Testa G., Giannelli S., Leonarduzzi G., Gamba P. (2022). ApoE3 vs. ApoE4 Astrocytes: A Detailed Analysis Provides New Insights into Differences in Cholesterol Homeostasis. Antioxidants.

[73] Zhang J., Liu Q. (2015). Cholesterol metabolism and homeostasis in the brain. Protein Cell.

[74] Li F., Chen Y., Anton M., Nielsen J. (2023). GotEnzymes: an extensive database of enzyme parameter predictions. Nucleic Acids Res..

[75] Li Z., Liu J. (2024). Thyroid dysfunction and Alzheimer's disease, a vicious circle. Front. Endocrinol..

[76] Uhlén M., Fagerberg L., Hallström B.M., Lindskog C., Oksvold P., Mardinoglu A., Sivertsson Å., Kampf C., Sjöstedt E., Asplund A. (2015). Proteomics. Tissue-based map of the human proteome. Science.

[77] Zhang X., Bao G., Liu D., Yang Y., Li X., Cai G., Liu Y., Wu Y. (2021). The Association Between Folate and Alzheimer's Disease: A Systematic Review and Meta-Analysis. Front. Neurosci..

[78] Naz N., Naqvi S.F., Hohn N., Whelan K., Littler P., Roncaroli F., Robinson A.C., Miyan J.A. (2023). Cerebral Folate Metabolism in Post-Mortem Alzheimer’s Disease Tissues: A Small Cohort Study. Int. J. Mol. Sci..

[79] Cook N., Yang C., Zeng Y., Sivasankaran S.K., Song S., Talozzi L., Western D., Yang C., Liu Y., Le Guen Y. (2025). Integrative Genetic, Proteogenomic, and Multi-omics Analyses Reveal Sex-Biased Causal Genes and Drug Targets in Alzheimer’s Disease. medRxiv.

[80] Chitkara S., Gonzalez A., Shah A.K., Shah A.K., Tappia P.S., Dhalla N.S. (2024). Hydrophilic Vitamins in Health and Disease.

[81] Zhang Z.-H., Cao X.-C., Peng J.-Y., Huang S.-L., Chen C., Jia S.-Z., Ni J.-Z., Song G.-L. (2022). Reversal of Lipid Metabolism Dysregulation by Selenium and Folic Acid Co-Supplementation to Mitigate Pathology in Alzheimer’s Disease. Antioxidants.

[82] Kim H.-J., Park J., Kim Y.S., Park J. (2021). The sex-specific effect of the apolipoprotein E allele and methylenetetrahydrofolate reductase gene polymorphism on the biochemical, anatomical, and cognitive profiles of patients clinically diagnosed with probable Alzheimer's disease. Int. J. Geriatr. Psychiatry.

[83] Lirong W., Mingliang Z., Mengci L., Qihao G., Zhenxing R., Xiaojiao Z., Tianlu C. (2022). The clinical and mechanistic roles of bile acids in depression, Alzheimer's disease, and stroke. Proteomics.

[84] Bell S.M., Barnes K., Clemmens H., Al-Rafiah A.R., Al-ofi E.A., Leech V., Bandmann O., Shaw P.J., Blackburn D.J., Ferraiuolo L., Mortiboys H. (2018). Ursodeoxycholic Acid Improves Mitochondrial Function and Redistributes Drp1 in Fibroblasts from Patients with Either Sporadic or Familial Alzheimer's Disease. J. Mol. Biol..

[85] Lopez-Ibañez J., Pazos F., Chagoyen M. (2023). MBROLE3: improved functional enrichment of chemical compounds for metabolomics data analysis. Nucleic Acids Res..

[86] Heirendt L., Arreckx S., Pfau T., Mendoza S.N., Richelle A., Heinken A., Haraldsdóttir H.S., Wachowiak J., Keating S.M., Vlasov V. (2019). Creation and analysis of biochemical constraint-based models using the COBRA Toolbox v.3.0. Nat. Protoc..

[87] McQuade A., Coburn M., Tu C.H., Hasselmann J., Davtyan H., Blurton-Jones M. (2018). Development and validation of a simplified method to generate human microglia from pluripotent stem cells. Mol. Neurodegener..

[88] Barrett T., Wilhite S.E., Ledoux P., Evangelista C., Kim I.F., Tomashevsky M., Marshall K.A., Phillippy K.H., Sherman P.M., Holko M. (2013). NCBI GEO: archive for functional genomics data sets—update. Nucleic Acids Res..

[89] Smid M., Coebergh van den Braak R.R.J., van de Werken H.J.G., van Riet J., van Galen A., de Weerd V., van der Vlugt-Daane M., Bril S.I., Lalmahomed Z.S., Kloosterman W.P. (2018). Gene length corrected trimmed mean of M-values (GeTMM) processing of RNA-seq data performs similarly in intersample analyses while improving intrasample comparisons. BMC Bioinf..

[90] Love M.I., Huber W., Anders S. (2014). Moderated estimation of fold change and dispersion for RNA-seq data with DESeq2. Genome Biol..

[91] Düz E., Çakır T. (2024). Effect of RNA-Seq data normalization on protein interactome mapping for Alzheimer’s disease. Comput. Biol. Chem..

[92] Lüleci H.B., Uzuner D., Cesur M.F., İlgün A., Düz E., Abdik E., Odongo R., Çakır T. (2024). A benchmark of RNA-seq data normalization methods for transcriptome mapping on human genome-scale metabolic networks. npj Syst. Biol. Appl..

[93] Landgraf A.J., Lee Y. (2020). Dimensionality reduction for binary data through the projection of natural parameters. J. Multivariate Anal..

[94] Wang M., Zhao Y., Zhang B. (2015). Efficient Test and Visualization of Multi-Set Intersections. Sci. Rep..

[95] Vijaymeena M., Kavitha K. (2016). A survey on similarity measures in text mining. Mach. Learn. Appl..

