# Supplementary material for: Genome-scale metabolic modeling uncovers cell-type specific signatures associated with APOE variants
Source: iScience. 2026 May 7;29(5):115638. doi: 10.1016/j.isci.2026.115638 (PMC13185917; doi:10.1016/j.isci.2026.115638)
Supplement: Document S1. Figures S1–S3 and Technical Note S1 [file mmc1.pdf]

iScience, Volume *29*

Supplemental information

Genome-scale metabolic modeling uncovers cell-type specific signatures associated with APOE variants

Dilara Uzuner Odongo, Roxan A. Stephenson, Linling Cheng, Linda G. Yang, Priyanka S. Narayan, Tunahan Çakır, and Madhav Thambisetty

Technical Note S1: Statistical Validation of Metabolic Perturbation Criteria

To ensure the 3-1 and 2-0 iMAT criteria did not inflate the number of detected differences, we tested our findings based on two criteria.

Genotype-Specificity: To evaluate if the results are genotype-specific, we performed permutation testing for each comparison by reassigning genotype labels across samples and enumerating all possible label combinations. Specifically, in the APOE3 vs APOE4 comparison of neuron cells, six samples were available (three in each group). We tested all possible permutations of genotype labels across the samples, leading to 10 alternatives including the original labeling. For each permuted case, we repeated our perturbed reaction selection criteria (3-1, 2-0, 3-0). We calculated the overlap of the identified reactions with the original results in each permuted case using the Jaccard index. Across all permuted configurations, the overlap with the original reaction set was consistently low (Jaccard index < 0.3), indicating that the identified reactions were not preserved under random genotype assignments. This suggests that the observed reaction patterns depend on the true APOE genotype grouping rather than arising from arbitrary sample combinations.

Biological Enrichment: To ensure the identified perturbed reaction sets (e.g., 439 reactions in neurons) were not random artifacts, we performed a Monte Carlo random selection analysis. For this, equal-sized sets from all reactions in Human-GEM were selected randomly (n=1000 iterations). For each iteration, we calculated the overlap between the randomly sampled set and the original perturbed reaction set. As a result, it was observed that over 96%, 99% and 99,5% of the randomly generated sets overlapped with fewer than 5% of the original reactions for neuron, astrocyte and microglia, respectively. Consequently, when testing the null hypothesis—defined as the probability of a random set overlapping with at least 5% of the original reaction set—the observed profiles showed statistically significant enrichment across all cell types. The resulting p-values for neurons (p=0.012), astrocytes (p=0.038), and microglia (p=0.005) confirm that these metabolic profiles represent distinct biological signatures rather than random artifacts.

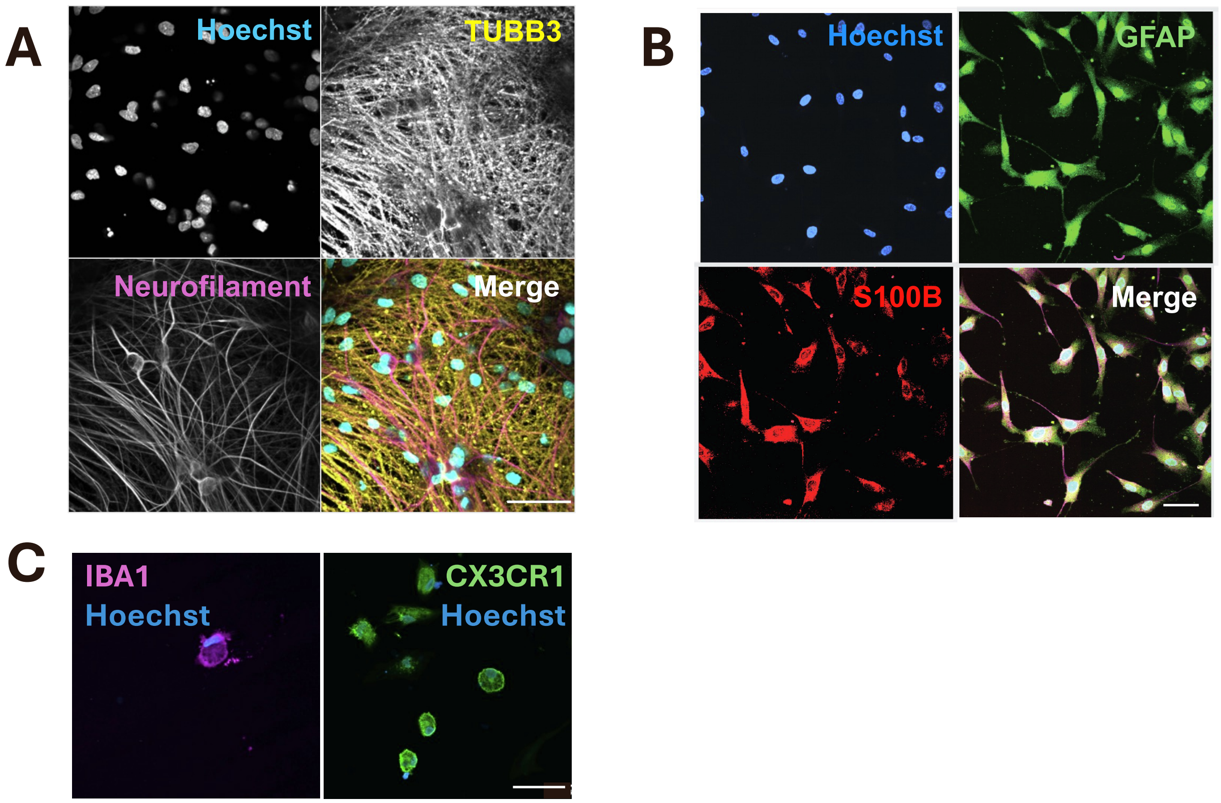

Figure S1: Immunocytochemistry of characteristic cell type-specific markers of iPSC-derived neurons **(A)**, astrocytes **(B)**, and microglia **(C)**, Related to STAR Methods

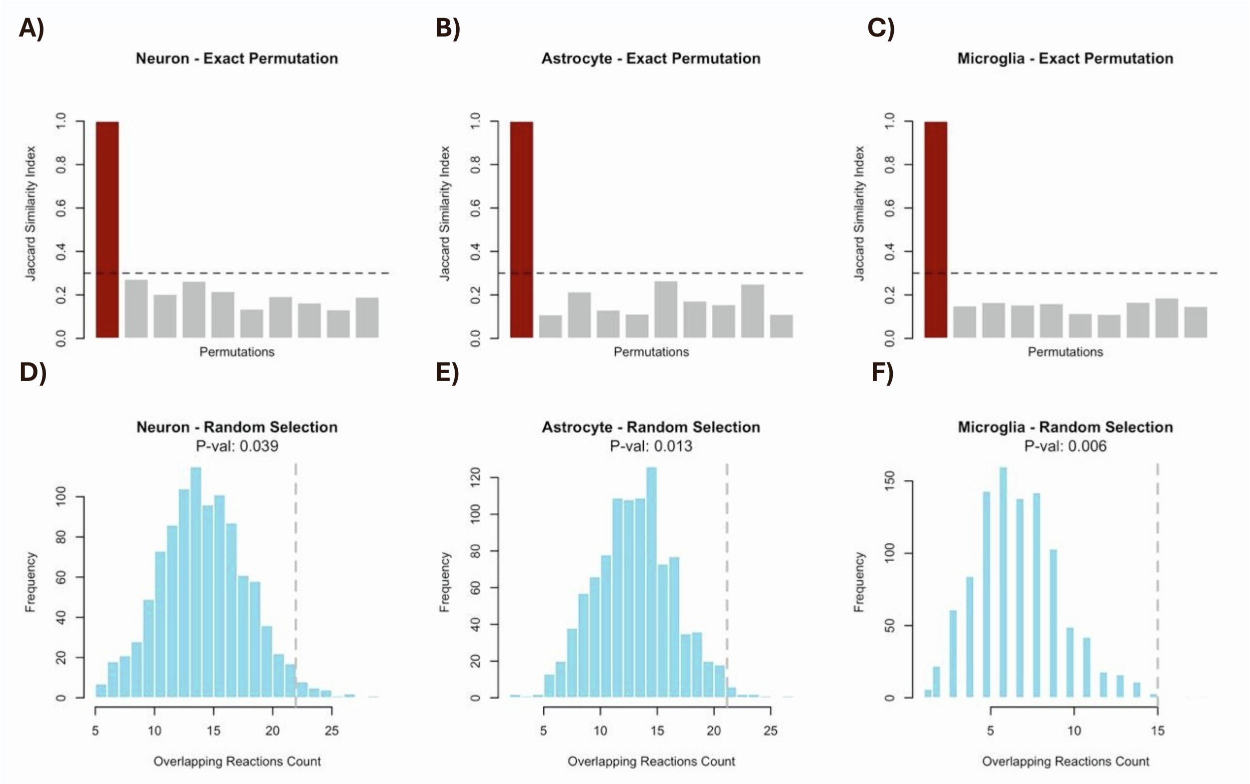

Figure S2. Robustness and Specificity Validation of Identified Metabolic Signatures, Related to Figure 1. (A-C) Exact Permutation Tests: Bar plots illustrate the Jaccard Similarity Index calculated for the perturbed reaction selections from all possible genotype label permutations to test the stability of the altered reaction sets. The horizontal dashed line marks a similarity threshold of 0.3. Bars colored in dark red correspond to the permutation that represents the original genotype labeling. **(D-F)** Monte Carlo Random Selection Analysis: Histograms display the null distribution of overlap counts generated from 1,000 randomly sampled reaction sets of equal size with original altered reactions from the Human-GEM model. The vertical dashed line marks the specific count corresponding to 5% of the original reaction set size.

A) iMAT metabolite-based pathway enrichment

B) Reporter metabolite pathway enrichment

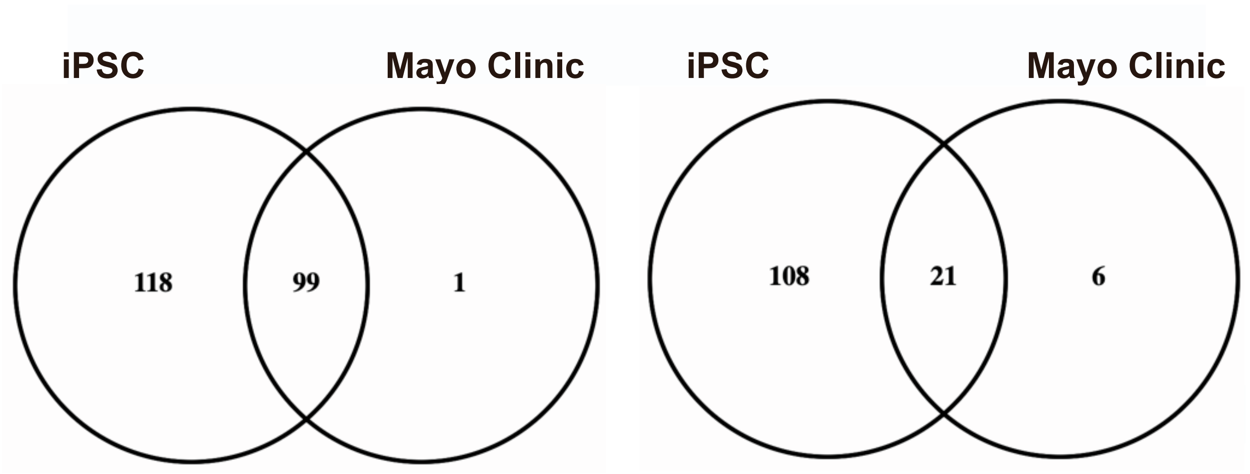

Figure S3: Comparison of Mayo Clinic dataset results with the iPSC dataset. RNA-seq data from the APOE3 and APOE4 genotype individuals in the Mayo Clinic dataset were compared with the iMAT approach and reporter metabolite approach, and metabolite-based pathway enrichment was applied. **A)** Venn diagram of the number of pathways enriched with the iMAT-derived metabolites. **B)** Venn diagram of the number of pathways enriched with the reporter metabolites.
